# Dietary L‐Tyrosine Sustains Egg Production and Quality by Modulating Promoter CpG Methylation, Gene Expression, and Hormone Profiles in Reproductive Signalling Pathways of Aged Laying Hens

**DOI:** 10.1111/jpn.70078

**Published:** 2026-06-07

**Authors:** Hasan Hüseyin İpçak

**Affiliations:** ^1^ Department of Animal Science, Faculty of Agriculture Dicle University Diyarbakır Türkiye

**Keywords:** DNA methylation, egg quality, gene expression, laying hens, laying performance, L‐tyrosine

## Abstract

Managing prolactin (PRL)‐mediated reproductive suppression is critical for maintaining egg production in commercial laying hens. L‐tyrosine (TYR), a precursor of dopamine (DA), may counteract age‐related reproductive decline by suppressing PRL, thereby sustaining productivity and egg quality in late‐phase laying hens. The present study evaluated the effects of dietary L‐tyrosine supplementation on laying performance, egg quality, reproductive hormone profiles, gene expression, and promoter CpG methylation in aged laying hens. A total of 180 Atak‐S laying hens (71 weeks old) were randomly assigned to three dietary treatments, each with six replicates of 10 hens, and were fed a basal diet without L‐tyrosine (control) or the basal diet supplemented with 0.5 g/kg (TYR0.5) or 1 g/kg (TYR1) L‐tyrosine for 12 weeks. Hens in the TYR1 group exhibited higher hen‐day egg production, improved feed conversion ratio, and significantly greater eggshell weight, shell thickness, Haugh unit, yolk index, and albumen index than the control group (*p* < 0.05). Dietary L‐tyrosine increased plasma DA and serum estradiol while reducing serum PRL (*p* < 0.05). L‐tyrosine also upregulated the expression of key dopaminergic and reproductive signalling genes, with corresponding promoter hypomethylation (*p* < 0.05), whereas PRL mRNA expression tended to decrease with concurrent promoter hypermethylation. A significant negative correlation was observed between target gene expression and promoter CpG methylation levels (*p* < 0.05). These findings indicate that dietary L‐tyrosine modulates reproductive hormone profiles and molecular responses within reproductive signalling pathways, resulting in improved laying performance and egg quality. Therefore, L‐tyrosine may represent a promising nutritional intervention to sustain reproductive function and productivity in late‐phase laying hens.

## 1 Introduction

Egg production is a major component of animal agriculture and represents a critical source of high‐quality protein for human nutrition. Eggs are nutrient‐dense foods that provide essential amino acids, vitamins, and minerals required for human health. Global egg production reached approximately 100 million tonnes in 2024, with hen eggs accounting for nearly 94% of total production (FAO 2024). Despite increasing demand, maintaining high laying performance and egg quality throughout the production cycle remains a major challenge, particularly in aged laying hens, where reproductive efficiency declines and economic profitability is reduced. In laying hens, egg production is tightly regulated by ovarian function and the hypothalamic–pituitary–gonadal (HPG) axis (Zhang et al. 2022). Gonadotropin‐releasing hormone stimulates the secretion of luteinizing hormone (LH) and follicle‐stimulating hormone (FSH), which regulate follicular maturation and ovulation. Estradiol (E2) plays a central role in coordinating these processes (Johnson 2014). Commercial laying hens typically reach peak production at 28–30 weeks of age, followed by a progressive decline to approximately 70%–75% by 72 weeks. This decline is largely associated with increased prolactin (PRL) secretion, which suppresses LH and FSH release, reduces circulating E2, and induces broody behaviour (Chaiseha and El Halawani 2005).

Nutritional strategies that modulate endocrine and metabolic pathways have been proposed to mitigate age‐related reproductive decline. Amino acids (AAs) play essential roles in maintaining laying performance, egg quality, and physiological function. Inadequate or imbalanced AA supply disrupts metabolic homeostasis and impairs reproductive performance and egg quality, particularly in late‐phase laying hens (Macelline et al. 2021; Uyanga et al. 2022). Previous studies have demonstrated that specific AAs can influence reproductive performance through endocrine and molecular mechanisms. For example, threonine supplementation improves egg production and feed efficiency (Azzam et al. 2017), serine enhances laying rate under low‐protein diets (Zhou et al. 2021), L‐arginine modulates hypothalamic and ovarian gene expression (Uyanga et al. 2022), and L‐leucine affects reproductive traits via mTOR signalling pathways (Motallebi et al. 2025).

Among AA‐related metabolic pathways, neurotransmitter regulation plays a key role in reproductive control. PRL secretion in laying hens is stimulated by hypothalamic vasoactive intestinal peptide (VIP), whereas dopamine (DA), derived from L‐3,4‐dihydroxyphenylalanine (L‐DOPA), acts as its primary inhibitor (Chokchaloemwong et al. 2015). During active laying, a balance between VIP and DA maintains low PRL concentrations and supports gonadotropin secretion, thereby facilitating ovulation. With advancing age, declining DA levels weaken this inhibitory control, leading to reduced gonadotropin secretion and decreased egg production (Chokchaloemwong et al. 2015). Several studies have shown that L‐DOPA supplementation restores catecholamine balance, suppresses VIP–PRL signalling, stimulates gonadotropin secretion, and improves laying performance (Tiwari and Chaturvedi 2003; Prasad et al. 2007; Omidiwura et al. 2020). L‐tyrosine, a semi‐essential AA and the primary dietary precursor of DA (Mao et al. 2018; İpçak and Kop Bozbey 2024), may therefore enhance dopaminergic activity and support reproductive hormone regulation in aged laying hens. Ali (2018) reported that L‐tyrosine supplementation improved egg number, egg weight, and shell thickness in post‐peak laying hens.

Nutritional epigenetic mechanisms also contribute to reproductive function. DNA methylation at CpG island promoters regulates transcription of genes involved in follicular development, steroidogenesis, and hormonal signalling (He et al. 2018; Wang and Ibeagha‐Awemu 2021). For instance, promoter CpG methylation of the estrogen receptor alpha (ESR1) affects gene expression during ovarian maturation (Guo et al. 2020), while dynamic methylation changes in reproductive biomarkers have also been reported throughout the laying cycle (Ponsuksili et al. 2025). Although increasing evidence links AA nutrition to reproductive physiology and epigenetic regulation, the role of dietary L‐tyrosine in sustaining laying performance and egg quality in aged laying hens remains unclear. In particular, it remains unknown whether L‐tyrosine can simultaneously modulate endocrine and molecular mechanisms. Therefore, it was hypothesised that dietary L‐tyrosine supplementation could improve egg production and quality by enhancing dopamine‐mediated suppression of prolactin and modulating reproductive signalling pathways at hormonal, transcriptional, and epigenetic levels. Accordingly, this study investigated the effects of dietary L‐tyrosine supplementation on laying performance, egg quality traits, hormone profiles, reproductive signalling–related gene expression, and promoter CpG methylation in aged laying hens.

## 2 Materials and Methods

### 2.1 Animals, Experimental Design, Diets and Housing

In this study, 180 Atak‐S laying hens (71 weeks old) were used from 71 to 82 weeks of age. Hens with similar egg yield and body weight were randomly assigned to three experimental groups, each with six replicates of 10 hens. The treatments included a control group (TYR0) receiving a basal diet without L‐tyrosine, while the other groups received basal diets supplemented with 0.5 g/kg (TYR0.5) or 1 g/kg (TYR1) L‐tyrosine. The experiment was conducted using a completely randomised design. Hens were housed in enriched group cages equipped with perches, nest boxes, and scratch pads, providing approximately 750 cm^2^ of floor area per hen. The facility was maintained at 22°C ± 2°C with a 16L:8D lighting programme. The purity of dietary L‐tyrosine (≥ 92.5%) was confirmed by LC‐Q‐TOF‐MS analysis (Agilent 6550 Technologies, Santa Clara, CA, USA) at EGE‐MATAL, Ege University (İzmir, Türkiye). All experimental diets were formulated in accordance with the Atak‐S management guidelines (TAEM 2015) and National Research Council NRC (1994) nutritional requirements for laying hens. The major feed ingredients and experimental diets were analysed for proximate composition using AOAC International (2000) official methods: crude protein (method 920.87), ether extract (method 920.39), crude fibre (method 962.09), crude ash (method 942.05), and dry matter (method 934.01). Total sugar (TS 12232) and starch (TS ISO 6493) contents were determined using standard colorimetric and polarimetric methods, respectively (Turkish Standards Institution TSE 1997, 2004). Diets in mash form and fresh water were supplied *ad libitum*. The detailed composition and nutrient profiles of the experimental diets are presented in Table 1.

**Table 1:** Ingredient composition (g/kg), nutrient content (%, as‐fed basis), and metabolizable energy (kcal/kg) of experimental diets for laying hens during the late‐production period.

Items	Experimental diets		
L‐tyrosine levels (g/kg)	0.0	0.5	1.0
Ingredients			
Corn	590.00	589.50	589.00
Soybean meal (46% CP)	210.00	210.00	210.00
Sunflower meal (32% CP)	50.00	50.00	50.00
Limestone (CaCO_3_)	95.00	95.00	95.00
Dicalciumphosphate	26.00	26.00	26.00
Sunflower oil (8800 Kcal/kg)	20.00	20.00	20.00
DL‐Methionine	2.50	2.50	2.50
Vitamin‐mineral premix^a^	2.50	2.50	2.50
NaCl	4.00	4.00	4.00
L‐tyrosine	—	0.50	1.00
Chemical composition analysed (%)			
Dry matter	90.70	90.80	90.85
Crude protein	16.40	16.45	16.38
Ether extract	3.69	3.58	3.65
Crude fibre	3.32	3.30	2.98
Crude ash	15.05	14.95	14.90
Starch	40.46	40.61	40.55
Sugar	3.12	3.18	3.15
Metabolizable energy (ME poultry), kcal/kg	2621.50	2622.19	2622.01
Calculated values (%)			
Lysine	0.83	0.83	0.83
Methionine+cystine	0.75	0.75	0.75
Calcium	4.30	4.30	4.30
Available phosphorus	0.55	0.55	0.55

^a^ Vitamin and mineral premix provided per kg of diet: vitamin A, 4,800,000 IU; vitamin D3, 1,000,000 IU; vitamin E, 12,000 mg; vitamin K3, 1600 mg; vitamin B1, 1200 mg; vitamin B2, 2800 mg; vitamin B6, 2000 mg; vitamin B12, 6 mg; CAL‐D pantothenate, 4000 mg; biotin, 18 mg; folic acid, 400 mg; canthaxanthin, 1000 mg; apo ester carotenoid, 200 mg; niacinamide, 1200 mg; choline chloride, 80,000 mg; Mn, 32,000 mg; Fe, 24,000 mg; Cu, 2000 mg; Zn, 24,000 mg; I, 400 mg; Co, 80 mg; Se, 60 mg; and CaCO_3_, 556,000 mg.

### 2.2 Performance Measurements

Body weights were measured individually at the beginning (71 weeks) and the end (82 weeks) of the trial. Eggs were collected, counted, and weighed daily. Hen‐day egg production (%), egg weight (g), egg mass (g/hen/day), feed intake (g/hen/day), and feed conversion ratio (FCR; g feed/g egg mass) were calculated on a replicate basis for the entire 12‐week trial (71–82 weeks) and for three consecutive 4‐week periods (71–74, 75–78, and 79–82 weeks). Mortality was recorded daily.

### 2.3 Sample Collection for Subsequent Analyses

A sample of 12 eggs (two per replicate) was collected at 74, 78, and 82 weeks of age for egg quality measurements. At 82 weeks, 12 hens per group (two hens per cage) were randomly selected, and blood samples were collected from the brachial vein using gentle manual restraint to minimise handling stress. Blood samples were centrifuged at 4000 rpm for 10 min to obtain serum and plasma for hormone analysis, while separate aliquots were used for RNA and genomic DNA extraction. Serum and plasma samples were stored at −20°C until analysis.

### 2.4 Egg Quality Measurements

For external quality assessment, eggs were individually weighed on a digital balance (Dikomsan, İzmir, Türkiye), and their length and width were determined with a digital caliper (Mitutoyo Corporation, Kanagawa, Japan). Egg specific gravity was measured using a precision balance (RADWAG AS 220.R2, Radom, Poland). The egg shape index was calculated according to Anderson et al. (2004) using the following formula: (egg width/egg length) × 100. After egg breaking, the eggshell weights were recorded after cleaning and drying, and the shell proportion was obtained using the formula: (shell weight/egg weight) × 100. Shell thickness, excluding membranes, was determined at three points with an electronic digital micrometre (Mitutoyo Corporation, Kanagawa, Japan). For internal quality assessment, the heights of albumen and yolk were determined with a mechanical pedestal micrometre (Accud, İstanbul, Türkiye). Albumen length, width, and yolk diameter were measured using a digital caliper (Mitutoyo Corporation, Kanagawa, Japan). Albumen and yolk indices were calculated as described by Heiman and Carver (1936) and Funk (1948): albumen index = [albumen height/((albumen length + albumen width)/2)] × 100, and yolk index = (yolk height/yolk diameter) × 100. The Haugh unit scores were calculated using the equation of Haugh (1937) as follows: 100 × log (albumen height + 7.57 − 1.7 × egg weight^0.37). Egg yolk colour was evaluated using a MiniScan colorimeter (3nh Technology Co. Ltd., Shenzhen, China), which provided L* (lightness), a* (redness), and b* (yellowness) values. Prior to measurement, the device was calibrated using standard tiles, and each sample was analysed in triplicate.

### 2.5 Determination of Blood Hormone Levels

Serum concentrations of steroid hormones, including estradiol (E2), testosterone (TESTO), and progesterone (PROG), as well as gonadotropins, including FSH, LH, and PRL, were determined using ELISA with commercial assay kits (Elabscience Biotechnology Co. Ltd., Wuhan, China) according to the manufacturer's protocols. The same ELISA method was also used to measure plasma corticosterone (CORT) and dopamine (DA) concentrations. All hormonal analyses were performed with a microplate reader (BioTek ELx800, Winooski, VT, USA) with assay‐specific wavelength settings. The coefficients of variation for the hormone assays were < 10% (intra‐assay) and < 15% (inter‐assay). Samples were analysed in duplicate, and the average values were used for statistical analysis.

### 2.6 Analysis of Gene Expression

Total RNA was isolated from blood samples with the High Pure RNA Kit (Roche Diagnostics, Basel, Switzerland) following the manufacturer's protocol. RNA concentration and purity were measured with a NanoDrop 2000 spectrophotometer (Thermo Scientific, Wilmington, DE, USA), and samples with A260/A280 ratios of 1.8–2.0 were included in subsequent analyses. RNA integrity was confirmed by agarose gel electrophoresis, which revealed clear 28S and 18S rRNA bands. cDNA synthesis was performed using the RevertAid RT Reverse Transcription Kit (Thermo Scientific, Wilmington, DE, USA). For each reaction, 1000 ng RNA per sample was combined with 0.5 µL random hexamer primer, 0.5 µL oligo dT primer, and nuclease‐free water, followed by incubation at 65°C for 5 min and then rapidly cooling on ice. Reverse transcription was carried out in a total reaction volume of 20 µL containing 1 µL RevertAid RT enzyme, 1 µL RiboLock RNase Inhibitor, 2 µL dNTP mix, and 4 µL 5X Reaction Buffer. The thermal profile consisted of incubation at 25°C for 5 min, cDNA synthesis at 42°C for 60 min, and enzyme inactivation at 70°C for 5 min. The synthesised cDNA was then stored at −20°C until use. qRT‐PCR analysis was conducted with a LightCycler 480 II system (Roche Diagnostics, Basel, Switzerland) using SYBR Green I Master‐Mix. The gene expression levels of *PRL*, *DRD1*, *ESR1*, *ESR2*, *FSHR*, and *LH* were quantified, with β‐actin (*ACTB*) used as the reference gene. Primer sequences for *Gallus gallus* were designed using the NCBI and ENSEMBL databases and verified by PRIMER BLAST to ensure specificity (Table 2). The qRT‐PCR reaction was set up in a 20 µL volume containing 3 µL cDNA, 10 µL SYBR Green Master Mix, 0.6 µL of each primer (10 µM), and 5.8 µL of nuclease‐free water. The cycling conditions were as follows: initial denaturation at 95°C for 5 min, followed by 45 cycles at 95°C for 10 s, 57°C for 15 s, and 72°C for 10 s. Amplification specificity was verified through melting curve analysis by gradually increasing the temperature from 60°C to 97°C. Data were analysed using the relative quantification method, with fold‐change values calculated as described by Lee et al. (2006). Target gene Ct values were normalised to the reference gene (*ACTB*), and relative expression was determined by comparison with that of the control group.

**Table 2:** Primer sequences for qRT‐PCR assessment of relative mRNA transcripts in aged laying hens.

Genes^a^	Accession number	Primer sequence (5′‐3′)	Primer position	Product length (bp)
*PRL*	NM_205466.3	F: CTGGAAGGCTGTAGAGATTGAG R: CATTTCCAGCATCACCAGAATG	474‐495 568‐547	95
*DRD1*	NM_001144848.3	F: GGATGTATGCCATCTCCTCTTC R: GAGCAATCCGGTATATCCTTGT	580‐601 669‐648	90
*ESR1*	NM_205183.2	F: TCAGCTCCTCCTTATCCTCTC R: GTCGTAGAGCGGAACTACATTT	1695‐1715 1794‐1773	100
*ESR2*	NM_001396358.1	F: TGCTCTGGTGTGGGTTATTG R: CAGATGCTCCATGCCCTTATTA	1635‐1654 1758‐1737	124
*FSHR*	NM_205079.2	F: TGAGGCAAACTTCACCTATCC R: TGCTGGAGACATGCTACATATT	918‐938 1014‐993	97
*LH*	NM_204936.2	F: ATGAGGAAACCAGCTAGTGAAG R: GTTGAGTAGCTGTAAGGGAAGG	1017‐1038 1141‐1120	125
*ACTB*	NM_205518.1	F:TGGGCCAGAAAGACAGCTAC R: CCGTGTTCAATGGGGTACTT	208‐227 289‐270	82

^a^ *PRL*, prolactin; *DRD1*, dopamine receptor D1; *ESR1*, estrogen receptor alpha; *ESR2*, estrogen receptor beta; *FSHR*, follicle‐stimulating hormone receptor; *LH*, luteinizing hormone; *ACTB*, β‐actin. F, forward; R, reverse.

### 2.7 DNA Methylation Analysis

Genomic DNA extraction from blood samples was performed with the High Pure PCR Template Preparation Kit (Roche Diagnostics, Basel, Switzerland) following the manufacturer's protocol. DNA concentration and purity were evaluated with a NanoDrop 2000 (Thermo Scientific, Wilmington, DE, USA), and samples showing an A260/A280 ratio in the range of 1.8–2.0 were used in downstream applications. DNA bisulfite conversion was conducted with the EZ DNA Methylation‐Gold™ Kit (Zymo Research, Irvine, CA, USA), as described in the supplier's protocol. For bisulfite treatment, 20 µL of DNA (500 pg–2 µg) was reacted with 130 µL CT Conversion Reagent and processed in a thermal cycler (Veriti 96‐Well, Applied Biosystems, USA) under conditions of 98°C for 10 min and 64°C for 2.5 h. Converted DNA was subsequently purified with Zymo‐Spin™ IC Columns, eluted in 10 µL of M‐Elution Buffer, and preserved at −20°C until analysis. Methylation‐specific qPCR (MSP‐qPCR) was conducted using a Roche LightCycler® 480 II system and SYBR Green I Master‐Mix (Roche Diagnostics, Basel, Switzerland). Primer pairs specific for methylated (M) and unmethylated (UM) CpG islands in the promoters of the target genes (*PRL*, *DRD1*, *ESR1*, *ESR2*, *FSHR*, and *LH*) were designed using MethPrimer software (https://methprimer.com/) based on sequences retrieved from the NCBI and ENSEMBL databases (Table 3). The MSP‐qPCR reaction was set up in a 10 μL volume containing 2 μL bisulfite‐converted DNA, 5 μL SYBR Green Master‐Mix, 2.6 μL nuclease‐free water, and 0.2 μL of each primer (10 μM). The cycling conditions consisted of initial denaturation at 95°C for 5 min, then 50 cycles of denaturation (95°C, 10 s), annealing (58°C, 15 s), and extension (72°C, 15 s), with a final melting curve analysis from 63°C to 97°C. The methylation status was determined by analysing the melting curve differences between methylated and unmethylated primer sets using the LightCycler 480 software in Tm‐Calling mode. CpGenome™ Universal Methylated and Unmethylated DNA Standards (Merck Millipore, Burlington, MA, USA) were used as positive controls in every PCR reaction. The methylation percentage was calculated based on the differential melting temperatures of the methylated and unmethylated products.

**Table 3:** Primers for MSP‐qPCR to assess DNA methylation in aged laying hens.

Genes^a^	Primer type^b^	Primer sequence (5′‐3′)	Product length (bp)
*PRL*	M_Forward_ M_Reverse_	GATAATTGGAGTTTTTAAGGATTAGC TTTTACATACATTCAACAAATCGTA	237
	UM_*Forward*_ UM_*Reverse*_	TGATAATTGGAGTTTTTAAGGATTAGTG TTTTACATACATTCAACAAATCATA	238
*DRD1*	M_Forward_ M_Reverse_	GTATACGGGATTATTTTGTTTCGTC AATACCGATACTTACTTTACGCGTC	108
	UM_Forward_ UM_Reverse_	ATATGGGATTATTTTGTTTTGTTGA AATACCAATACTTACTTTACACATC	106
*ESR1*	M_Forward_ M_Reverse_	TTAGTTTTTTTGGTTTGATTTTAGC CCAAAAACTCTTACCTTATACCGAC	194
	UM_Forward_ UM_Reverse_	GGATTAGTTTTTTTGGTTTGATTTTAGT CAAAAACTCTTACCTTATACCAAC	196
*ESR2*	M_Forward_ M_Reverse_	GTAGTGGTTTTAACGTATCGTATCG AAAAAAATTTTACCTTTCAAACGAA	174
	UM_Forward_ UM_Reverse_	TAGTGGTTTTAATGTATTGTATTGG AAAAAAATTTTACCTTTCAAACAAA	173
*FSHR*	M_Forward_ M_Reverse_	TTTATTAGAATAGTGGTTGTTTCGA ATATTACAACTCCCATTCACATCGT	114
	UM_Forward_ UM_Reverse_	GTTTATTAGAATAGTGGTTGTTTTGA ATATTACAACTCCCATTCACATCATT	115
*LH*	M_Forward_ M_Reverse_	TGGTGTTATTCGCGAATTTAAC TAAAACGTACTCGACCCTCG	208
	UM_Forward_ UM_Reverse_	TTGGTGTTATTTGTGAATTTAATGA TAAAACATACTCAACCCTCA	209

^a^ *PRL*, prolactin; *DRD1*, dopamine receptor D1; *ESR1*, estrogen receptor alpha; *ESR2*, estrogen receptor beta; *FSHR*, follicle‐stimulating hormone receptor; *LH*, luteinizing hormone.

^b^ M, Methylation‐specific primers; UM, Unmethylation‐specific primers.

### 2.8 Statistical Analysis

All experimental data were analysed using IBM SPSS Statistics for Windows (Version 22.0; IBM Corp., Armonk, NY, USA 2013). The Shapiro–Wilk test was used to assess the normality of the data. Normally distributed variables were analysed by one‐way ANOVA, and Duncan's test was applied to identify significant differences. Linear and quadratic trends in response to dietary L‐tyrosine levels were evaluated using orthogonal polynomial contrast. Owing to the non‐normal distribution of methylation data, the Kruskal–Wallis test was applied, and pairwise differences were further examined using the Mann–Whitney U test. Mortality data were evaluated using the chi‐square test. Correlations between gene expression levels and DNA methylation patterns were evaluated using Pearson's correlation analysis, and determination coefficients (r^2^) were calculated. Statistical significance was defined as *p* < 0.05, whereas 0.05 < *p* < 0.10 was regarded as a tendency. Data are expressed as mean ± SEM.

## 3 Results

### 3.1 Laying Performance

The effects of dietary L‐tyrosine supplementation on laying performance in aged laying hens are presented in Table 4. Hen‐day egg production was higher in hens fed 1 g/kg L‐tyrosine during weeks 75–78 (71.18% vs. 76.19% for TYR0 vs. TYR1, *p* < 0.05). Over the entire period, egg production increased from 71.92% in the control to 75.53% in the TYR1 group (*p* < 0.05). Egg weight showed a quadratic response, with the highest value observed in the TYR0.5 group (65.49 g vs. 63.65 g in the control, *p* < 0.05). Egg mass was greatest in the TYR0.5 group over the entire period (*p* < 0.05). Feed intake decreased linearly with increasing L‐tyrosine inclusion (*p* < 0.05). FCR was improved in both supplemented groups, with final values of 2.37 and 2.33 in TYR0.5 and TYR1 groups compared with 2.51 in the control, representing improvements of 5.6% and 7.2%, respectively (*p* < 0.05). L‐tyrosine supplementation did not affect mortality (*p* > 0.05).

**Table 4:** Effect of dietary L‐tyrosine inclusion levels on productive and laying performance of aged laying hens (*n *= 6).

Item	Dietary treatment^a^			Pooled SEM^b^	*p‐*values*		
Supplemental TYR, g/kg	0.0	0.5	1.0		Main	Linear	Quadratic
Initial BW, g	1871.75	1864.82	1866.79	15.290	0.972	0.896	0.891
Final BW, g	1969.70	1960.68	1965.71	28.785	0.981	0.932	0.862
Hen‐day egg production, %							
71 to 74 wk	74.76	76.19	76.67	1.063	0.753	0.471	0.835
75 to 78 wk	71.18^b^	75.28^ab^	76.19^a^	0.907	0.048	0.020	0.407
79 to 82 wk	70.47	71.07	73.56	1.195	0.539	0.297	0.710
Total (71 to 82 wk)	71.92^b^	73.79^ab^	75.53^a^	0.629	0.044	0.013	0.911
Egg weight, g							
71 to 74 wk	63.46^b^	65.10^a^	63.04^b^	0.330	0.023	0.596	0.007
75 to 78 wk	63.92^b^	65.98^a^	63.80^b^	0.276	0.001	0.846	< 0.001
79 to 82 wk	62.78^b^	65.42^a^	63.26^b^	0.254	< 0.001	0.374	< 0.001
Total (71 to 82 wk)	63.65^b^	65.49^a^	63.37^b^	0.171	< 0.001	0.475	< 0.001
Egg mass, g/hen/d							
71 to 74 wk	47.55	49.70	48.31	0.743	0.494	0.678	0.267
75 to 78 wk	46.02	47.44	48.60	0.613	0.229	0.088	0.913
79 to 82 wk	44.61	46.78	46.52	0.796	0.522	0.349	0.469
Total (71 to 82 wk)	46.12^b^	48.60^a^	47.91^ab^	0.423	0.047	0.082	0.079
Feed intake, g/hen/d							
71 to 74 wk	114.97	112.43	111.32	0.666	0.078	0.027	0.604
75 to 78 wk	115.00	113.37	112.18	0.595	0.154	0.055	0.862
79 to 82 wk	116.41^a^	114.29^ab^	110.69^b^	0.786	0.007	0.002	0.649
Total (71 to 82 wk)	116.05^a^	114.47^b^	112.01^c^	0.344	< 0.001	< 0.001	0.530
Feed conversion ratio							
71 to 74 wk	2.44	2.25	2.31	0.035	0.079	0.135	0.092
75 to 78 wk	2.52^a^	2.38^b^	2.30^b^	0.031	0.009	0.002	0.622
79 to 82 wk	2.62^a^	2.45^b^	2.38^b^	0.036	0.021	0.007	0.454
Total (71 to 82 wk)	2.51^a^	2.37^b^	2.33^b^	0.020	0.001	< 0.001	0.219
Mortality, %	2.08	—	2.08	—	0.602	—	—

*Note:* The values are means of six replicates per treatment. Within each row, mean values indicated by different superscript letters differ significantly (*p < 0.05).

^a^ Dietary treatment groups included: basal diet without L‐tyrosine (TYR0; control) and basal diet supplemented with 0.5 and 1.0 g L‐tyrosine/kg (TYR0.5 and TYR1, respectively).

^b^ Pooled standard error of the mean.

### 3.2 External Egg Quality

The effects of dietary L‐tyrosine supplementation on external egg quality are given in Table 5. Eggshell weight increased with L‐tyrosine inclusion, with the TYR1 group recording 7.8% greater shell weight than the control at 78 weeks (7.30 vs. 6.77 g, *p* < 0.05). Eggshell thickness also increased at 78 weeks, reaching 0.369 mm in TYR1 compared with 0.343 mm in the control (linear effect, *p* < 0.05). Egg specific gravity, eggshell proportion, and shape index were not affected by L‐tyrosine supplementation at any sampling age (*p* > 0.05).

**Table 5:** Effect of dietary L‐tyrosine inclusion levels on external egg quality in aged laying hens (*n* = 12).

Item	Dietary treatment^a^			Pooled SEM^b^	*p‐*values*		
Supplemental TYR, g/kg	0.0	0.5	1.0		Main	Linear	Quadratic
Egg specific gravity							
74 wk	1.078	1.077	1.079	0.001	0.887	0.734	0.721
78 wk	1.072	1.075	1.074	0.001	0.367	0.290	0.347
82 wk	1.074	1.077	1.076	0.002	0.286	0.244	0.291
Egg shape index, %							
74 wk	75.93	76.58	76.46	0.307	0.658	0.490	0.557
78 wk	76.26	75.48	75.83	0.300	0.579	0.563	0.385
82 wk	75.18	75.09	75.69	0.343	0.758	0.558	0.637
Eggshell weight, g							
74 wk	7.11	7.05	7.26	0.099	0.683	0.537	0.533
78 wk	6.77^b^	6.99^ab^	7.30^a^	0.077	0.020	0.005	0.802
82 wk	7.01	7.22	7.20	0.109	0.689	0.478	0.634
Eggshell proportion, %							
74 wk	11.94	11.82	11.72	0.164	0.864	0.591	0.978
78 wk	11.09	11.51	11.43	0.104	0.215	0.186	0.247
82 wk	11.51	11.96	12.02	0.175	0.435	0.246	0.589
Eggshell thickness, mm							
74 wk	0.346	0.345	0.342	0.004	0.942	0.733	0.969
78 wk	0.343	0.356	0.369	0.005	0.127	0.043	0.981
82 wk	0.322	0.335	0.313	0.005	0.246	0.488	0.125

*Note:* Within each row, mean values indicated by different superscript letters differ significantly (*p < 0.05).

^a^ Dietary treatment groups included: basal diet without L‐tyrosine (TYR0; control) and basal diet supplemented with 0.5 and 1.0 g L‐tyrosine/kg (TYR0.5 and TYR1, respectively).

^b^ Pooled standard error of the mean.

### 3.3 Internal Egg Quality

The effects of dietary L‐tyrosine supplementation on internal egg quality are shown in Table 6. The albumen index increased at 82 weeks, with the TYR1 group recording 21.8% higher values than the control (9.79% vs. 8.04%, *p* < 0.05). The yolk index was elevated in L‐tyrosine–supplemented groups across all sampling periods (*p* < 0.05), with the greatest difference observed in the TYR1 group at 82 weeks (46.99% vs. 43.25% in the control, *p* < 0.05). Haugh unit scores were significantly higher in the TYR1 group at 74 weeks (86.68 vs. 80.68 in the control, *p*_*lin*_ < 0.05) and 82 weeks (88.09 vs. 81.76 in the control, *p* < 0.05). Yolk colour parameters (L*, a*, and b*) were not affected by L‐tyrosine supplementation at any sampling age (*p* > 0.05).

**Table 6:** Effect of dietary L‐tyrosine inclusion levels on internal egg quality in aged laying hens (*n* = 12).

Item	Dietary treatment^a^			Pooled SEM^b^	*p‐*values*		
Supplemental TYR, g/kg	0.0	0.5	1.0		Main	Linear	Quadratic
Albumen index, %							
74 wk	8.30	8.92	9.30	0.241	0.238	0.095	0.817
78 wk	8.65	8.84	8.75	0.164	0.901	0.816	0.696
82 wk	8.04^b^	8.12^b^	9.79^a^	0.301	0.028	0.018	0.195
Yolk index, %							
74 wk	42.80^b^	45.37^a^	44.91^a^	0.363	0.007	0.014	0.038
78 wk	45.54^b^	47.28^a^	46.48^ab^	0.295	0.045	0.160	0.037
82 wk	43.25^b^	44.54^b^	46.99^a^	0.471	0.002	0.001	0.536
Haugh unit							
74 wk	80.68^b^	83.82^ab^	86.68^a^	0.946	0.033	0.010	0.943
78 wk	83.29	83.25	84.23	0.593	0.755	0.523	0.692
82 wk	81.76^b^	81.98^b^	88.09^a^	1.375	0.046	0.028	0.222
Egg yolk colour L*							
74 wk	45.50	45.78	45.17	0.615	0.925	0.832	0.743
78 wk	47.67	46.36	48.18	0.432	0.221	0.660	0.095
82 wk	49.39	48.51	50.55	0.403	0.142	0.195	0.137
Egg yolk colour a*							
74 wk	11.38	10.04	10.25	0.318	0.188	0.151	0.252
78 wk	9.23	8.76	8.43	0.213	0.309	0.131	0.888
82 wk	12.25	11.42	12.07	0.328	0.602	0.827	0.340
Egg yolk colour b*							
74 wk	26.91	26.13	26.65	0.373	0.700	0.783	0.428
78 wk	27.66	27.21	27.70	0.403	0.871	0.983	0.602
82 wk	29.33	28.36	29.67	0.598	0.703	0.786	0.432

*Note:* Within each row, mean values indicated by different superscript letters differ significantly (*p < 0.05).

^a^ Dietary treatment groups included: basal diet without L‐tyrosine (TYR0; control) and basal diet supplemented with 0.5 and 1.0 g L‐tyrosine/kg (TYR0.5 and TYR1, respectively).

^b^ Pooled standard error of the mean.

### 3.4 Blood Hormone Levels

**Figure 1:**
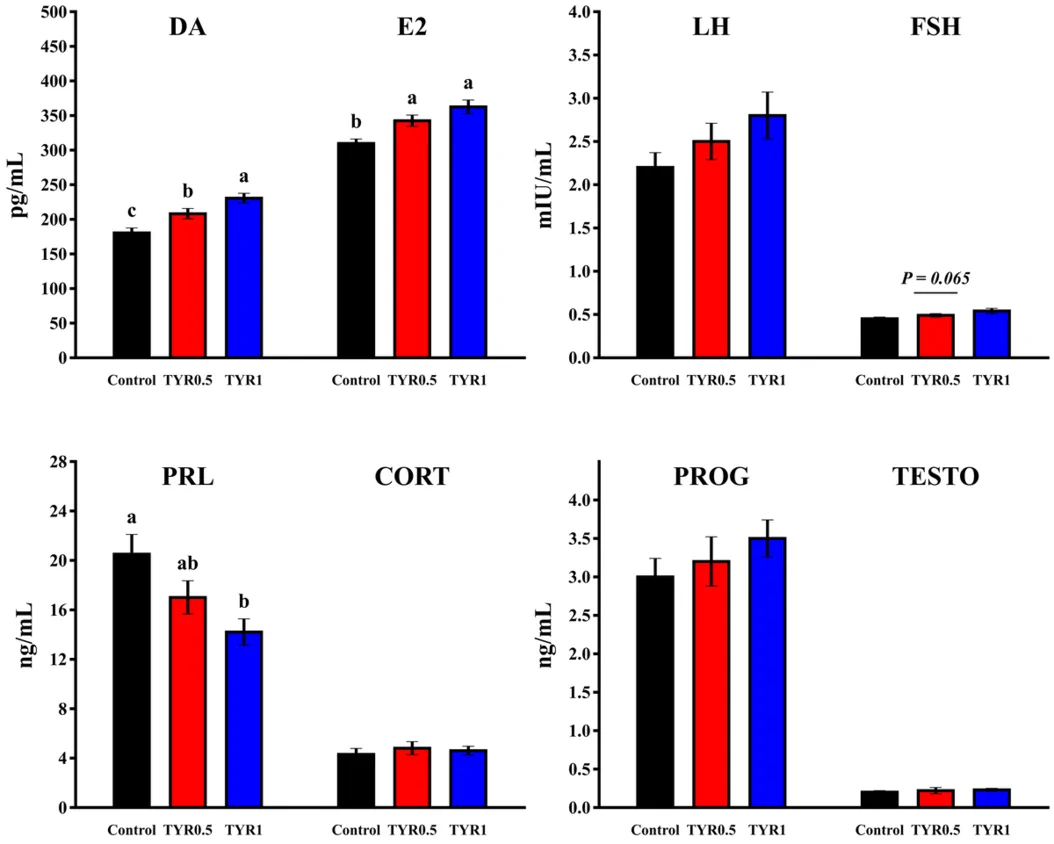
Effect of dietary L‐tyrosine inclusion levels on reproductive and stress‐related hormones in late‐phase laying hens. Values are means with SEM error bars. Letters indicate statistical differences at *p* < 0.05. Dietary treatment groups included: basal diet without L‐tyrosine (TYR0; control) and basal diet supplemented with 0.5 and 1.0 g L‐tyrosine/kg (TYR0.5 and TYR1, respectively). DA, Dopamine; E2, Estradiol; FSH, Follicle‐Stimulating Hormone; LH, Luteinizing Hormone; PRL, Prolactin; PROG, Progesterone; CORT, Corticosterone; TESTO, Testosterone. SEM, standard error of the mean, (*n* = 12). [Color figure can be viewed at wileyonlinelibrary.com]

The effects of dietary L‐tyrosine supplementation on reproductive and stress‐related hormone concentrations in aged laying hens are presented in Figure 1. Plasma DA concentrations increased dose‐dependently with L‐tyrosine inclusion, with the TYR1 group showing 27.8% greater values than the control (230.52 vs. 180.36 pg/mL, *p* < 0.05). Serum E2 concentrations were also significantly higher in TYR0.5 (342.45 pg/mL) and TYR1 (362.44 pg/mL) compared with the control (309.62 pg/mL, *p* < 0.05). Conversely, serum PRL levels decreased with increasing L‐tyrosine inclusion, being 30.7% lower in the TYR1 group than in the control (14.20 vs. 20.50 ng/mL, *p* < 0.05). FSH tended to increase with L‐tyrosine supplementation (*p* < 0.10), whereas LH, CORT, PROG, and TESTO were not affected by treatment (*p* > 0.05).

### 3.5 Gene Expression and Promoter CpG Methylation Patterns

**Figure 2:**
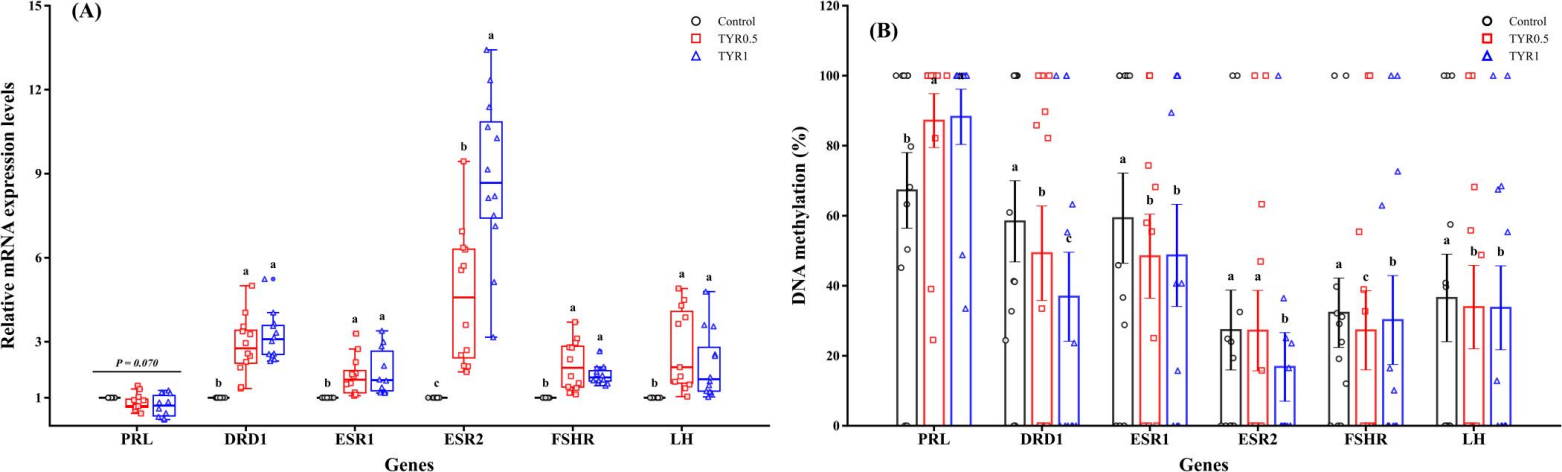
Effect of dietary L‐tyrosine inclusion levels on gene expression and DNA methylation status in late‐phase laying hens. (A) Relative mRNA expression levels and (B) DNA methylation percentages of reproductive signalling genes. Values are means with SEM error bars. Letters indicate statistical differences at *p* < 0.05. Dietary treatment groups included: basal diet without L‐tyrosine (TYR0; control) and basal diet supplemented with 0.5 and 1.0 g L‐tyrosine/kg (TYR0.5 and TYR1, respectively). *PRL*, prolactin; *DRD1*, dopamine receptor D1; *ESR1*, estrogen receptor alpha; *ESR2*, estrogen receptor beta; *FSHR*, follicle‐stimulating hormone receptor; *LH*, luteinizing hormone. SEM, standard error of the mean, (*n* = 12). [Color figure can be viewed at wileyonlinelibrary.com]

The expression profiles of genes involved in reproductive signalling pathways are given in Figure 2A. *DRD1* expression increased 2.86‐ and 3.19‐fold in the TYR0.5 and TYR1 groups (*p* < 0.05). *ESR1* expression was also upregulated (1.77‐ and 1.96‐fold, respectively), whereas *ESR2* showed the most pronounced response, increasing 4.61‐fold in TYR0.5 and 8.87‐fold in TYR1 (*p* < 0.05). *FSHR* expression increased 2.17‐ and 1.81‐fold, and *LH* expression was 2.77‐ and 2.20‐fold higher in the TYR0.5 and TYR1 groups, respectively, compared with the control (*p* < 0.05). In contrast, *PRL* expression tended to decrease with increasing dietary L‐tyrosine inclusion (*p* < 0.10).

The DNA methylation profiles of dopaminergic and reproductive genes are shown in Figure 2B. *PRL* promoter methylation increased, reaching 87.14% in the TYR0.5 group and 88.23% in the TYR1 group, compared with 67.22% in the control (*p* < 0.05). In contrast, *DRD1* methylation decreased from 58.37% in the control group to 49.27% and 36.85% in the TYR0.5 and TYR1 groups (*p* < 0.05). *ESR1* methylation declined from 59.28% to approximately 48% in both treatment groups (*p* < 0.05). *ESR2* methylation was lower in the TYR1 group than in the control (16.80% vs. 27.33%, *p* < 0.05)*. FSHR* and *LH* promoters also showed reduced methylation in both L‐tyrosine‐supplemented groups (*p* < 0.05).

### 3.6 Correlations Between Gene Expression and Promoter DNA Methylation

**Figure 3:**
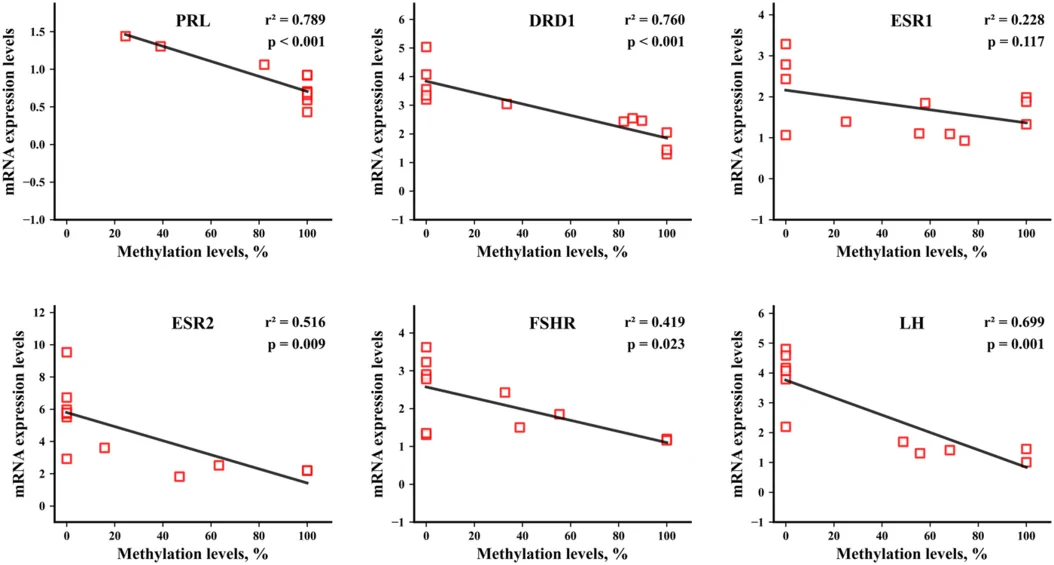
Correlations between mRNA expression levels and DNA methylation status of dopaminergic and reproductive genes in laying hens fed 0.5 g/kg L‐tyrosine (TYR0.5 group). *PRL*, prolactin; *DRD1*, dopamine receptor D1; *ESR1*, estrogen receptor alpha; *ESR2*, estrogen receptor beta; *FSHR*, follicle‐stimulating hormone receptor; *LH*, luteinizing hormone. r^2^, coefficient of determination from linear regression. *p* < 0.05, (*n* = 12). [Color figure can be viewed at wileyonlinelibrary.com]

The correlations between relative mRNA expression and promoter DNA methylation levels in the TYR0.5 group are presented in Figure 3. *PRL* expression was negatively correlated with promoter methylation (r = −0.888, *p* < 0.05). *DRD1* expression was also negatively correlated with methylation (r = −0.872, *p* < 0.05). *ESR1* expression was not associated with promoter methylation (r = −0.477, *p* > 0.05), whereas *ESR2* expression showed a significant negative correlation (r = −0.718, *p* < 0.05). *FSHR* and *LH* expression were also negatively correlated with promoter methylation (r = −0.647 and −0.836, respectively; *p* < 0.05).

**Figure 4:**
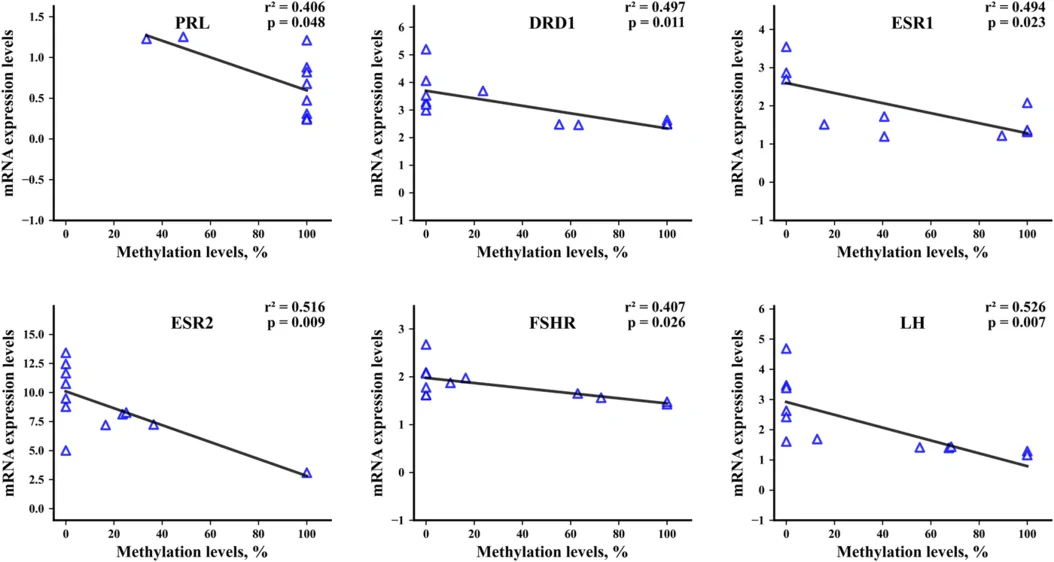
Correlations between mRNA expression levels and DNA methylation status of dopaminergic and reproductive genes in laying hens fed 1.0 g/kg L‐tyrosine (TYR1 group). *PRL*, prolactin; *DRD1*, dopamine receptor D1; *ESR1*, estrogen receptor alpha; *ESR2*, estrogen receptor beta; *FSHR*, follicle‐stimulating hormone receptor; *LH*, luteinizing hormone. r^2^, coefficient of determination from linear regression. *p* < 0.05, (*n* = 12). [Color figure can be viewed at wileyonlinelibrary.com]

Negative correlations between gene expression and promoter DNA methylation were similarly observed in the TYR1 group (Figure 4). All evaluated genes showed significant negative correlations with their respective promoter methylation levels (r = −0.637 to −0.727, *p* < 0.05). Overall, a consistent inverse relationship between promoter CpG methylation and mRNA expression was observed across all evaluated genes and treatment groups.

## 4 Discussion

Dietary L‐tyrosine supplementation significantly improved the laying performance of aged laying hens. Hen‐day egg production was 5.02% higher in the TYR1 group than in the control over the entire experimental period. Ali (2018) reported that tyrosine, alone or in combination with tryptophan, increased egg number in post‐peak layers, which is consistent with the present findings. Similar improvements in egg production have been reported with threonine (Jiang et al. 2019; Hossaninejad et al. 2021) and serine supplementation (Zhou et al. 2021). By contrast, isoleucine (Ullah et al. 2022) and optimised valine/lysine ratios (Nahavandinejad et al. 2024) did not influence egg production, reflecting the amino acid‐specific responses of laying performance. Unlike threonine and serine, which act primarily through protein anabolism and intestinal barrier function, the productive benefits of L‐tyrosine are most likely mediated through its role as the primary precursor of dopamine (David et al. 2003; Chokchaloemwong et al. 2015). Dopamine inhibits VIP‐stimulated prolactin secretion and maintains gonadotropin release, thereby supporting follicular development in late‐phase hens. As hens age, progressive declines in catecholamine levels impair this neuroendocrine balance, leading to elevated prolactin, suppressed gonadotropin secretion, and reduced ovulation frequency. L‐tyrosine supplementation may have restored dopaminergic tone, thereby mitigating the ovulatory decline characteristic of the late production phase. Egg weight showed a quadratic response to dietary L‐tyrosine. The TYR0.5 group produced the heaviest eggs throughout the study, exceeding the control by 2.9%, while the TYR1 group did not differ significantly from the control. Ali (2018) reported no effect of dietary tyrosine on egg weight, and methionine (Gao et al. 2024) and arginine (Sun et al. 2022) likewise did not affect egg weight. The observed quadratic response may be associated with dose‐dependent differences in albumen deposition, a major determinant of egg weight, likely reflecting variation in oviductal protein secretion across L‐tyrosine inclusion levels. Total egg mass was greatest in the TYR0.5 group throughout the study, which can be explained by the combined effects of increased egg weight and sustained laying rate, in line with results reported for valine (Jian et al. 2021) and isoleucine supplementation (Ullah et al. 2022). Feed intake decreased linearly with increasing L‐tyrosine inclusion, while FCR improved by 5.6% and 7.2% in TYR0.5 and TYR1, respectively. Similar improvements in FCR, without changes in feed intake, have been reported with threonine supplementation (Hossaninejad et al. 2021) and optimised valine/lysine ratios (Nahavandinejad et al. 2024). The reduction in feed intake together with improved FCR suggests enhanced nutrient utilisation efficiency rather than increased feed intake. This effect may reflect dopamine‐mediated adjustments in hypothalamic energy allocation, as dopaminergic pathways can influence energy partitioning toward reproductive functions in avian species (Mao et al. 2018).

L‐tyrosine supplementation also improved multiple egg quality parameters in aged laying hens. Eggshell weight was 7.83% higher and shell thickness was 7.58% greater in the TYR1 group than in the control at 78 weeks. Ali (2018) reported improved shell thickness with tyrosine in post‐peak layers, supporting the present findings. Methionine enhanced eggshell mineralisation through increased synthesis of sulphur‐containing matrix proteins (Gao et al. 2024), while valine reduced shell weight without affecting thickness (Wen et al. 2019) and arginine had no significant effect on shell traits (Yang et al. 2016). The improvement in shell quality in the present study is most likely attributable to the endocrine effects of L‐tyrosine rather than direct involvement in shell matrix protein metabolism. Elevated estradiol concentrations in supplemented hens may promote the expression of calcium‐binding proteins in the shell gland and enhance carbonic anhydrase activity, both of which facilitate calcium carbonate deposition during shell formation (Chokchaloemwong et al. 2015). In addition, the reduction in serum prolactin in the TYR1 group may have relieved the suppression of shell gland secretory function commonly associated with hyperprolactinemia during the late laying phase, thereby supporting more efficient calcification. The Haugh unit was significantly higher in the TYR1 group at 74 and 82 weeks, and the albumen index improved by 21.77% at 82 weeks in the same group. Arginine supplementation improved albumen quality through enhanced oviduct function and antioxidant capacity (Sun et al. 2022), and L‐leucine improved albumen traits through mTOR‐mediated protein synthesis (Motallebi et al. 2025). Nahavandinejad et al. (2024) reported improved Haugh unit scores with optimised valine/lysine ratios, while higher dietary valine reduced the Haugh unit despite increasing albumen weight (Wen et al. 2019), and tryptophan supplementation had no effect on albumen quality (Khattak and Helmbrecht 2019). These contrasting findings indicate that albumen quality responses are highly dependent on amino acid type and mode of action. In the present study, the reduction in circulating prolactin and the trend toward lower corticosterone in TYR1 may have contributed to albumen quality improvements by reducing glucocorticoid‐driven proteolysis of structural proteins in the magnum and alleviating stress‐related impairment of oviduct secretory activity. The yolk index was significantly higher in the TYR1 group at all sampling ages, with increases ranging from 2.06% to 8.65% relative to the control. Nahavandinejad et al. (2024) reported comparable yolk index improvements with optimised valine/lysine ratios, while decreased yolk index has been observed with valine and isoleucine supplementation (Wen et al. 2019; Ullah et al. 2022). The improvement in yolk index in the present study may be related to the stimulatory effect of elevated estradiol on hepatic vitellogenin synthesis and yolk precursor deposition, processes regulated by sustained estrogen receptor signalling in both the liver and ovary.

Serum dopamine was significantly elevated in the TYR1 group, in agreement with Mao et al. (2018), who demonstrated that increasing L‐DOPA availability raises hypothalamic dopamine concentrations and sustains reproductive hormone secretion in aged laying hens. Dopamine also modulates the hypothalamic‐pituitary‐adrenal axis (Hewlett et al. 2014), which may partly explain the observed trend toward lower corticosterone and improved stress resilience in the present study. Elevated dopamine has been shown to suppress VIP‐stimulated prolactin secretion (Rangel et al. 2009; Chokchaloemwong et al. 2015), which aligns with the reduced serum prolactin concentrations observed in the TYR1 group. Comparable reductions in prolactin have been reported following nutritional interventions in laying hens (Bana et al. 2021). The suppression of prolactin may relieve its inhibitory influence on GnRH neurons, which may account for the trend toward higher FSH concentrations observed in supplemented groups. Increases in FSH have also been reported with octacosanol (Long et al. 2017) and curcumin supplementation (Liu et al. 2020). Serum estradiol was elevated in both supplemented groups. Mao et al. (2018) showed that enhanced dopaminergic signalling stimulates estrogen synthesis in the ovarian stroma and pre‐hierarchical follicles, where D1 and D4 receptor mRNAs are enriched. Comparable estradiol elevations have been reported with fennel seed essential oil supplementation (İpçak et al. 2025), while astaxanthin had no significant effect on estradiol secretion (He et al. 2023), highlighting nutrient‐specific differences in endocrine responses. The elevated estradiol concentrations are further consistent with the upregulation of *ESR2* and its promoter hypomethylation in supplemented groups, suggesting that L‐tyrosine enhanced estrogenic activity through both increased ligand availability and epigenetic regulation of estrogen receptor gene expression. At the transcriptional level, *DRD1* expression was significantly higher in both L‐tyrosine–treated groups, consistent with previous findings (Mao et al. 2018). The upregulation of *DRD1* may enhance dopaminergic receptor signalling, thereby suppressing *PRL* transcription and potentially reinforcing HPG axis activity through a feedback mechanism linking dietary precursor availability to reproductive gene regulation. Both *ESR1* and *ESR2* were upregulated in supplemented groups, with *ESR2* reaching 8.87‐fold in TYR1 group. Guo et al. (2020) demonstrated that *ESR1* promoter methylation modulates receptor abundance and egg production, and Asiamah et al. (2021) reported that elevated *ESR2* expression improves follicular development and reproductive hormone profiles. This increase in circulating estradiol may have further amplified estrogen receptor expression through ligand‐driven transcriptional feedback (Abobaker et al. 2017), supporting estrogen‐mediated regulation of follicular maturation and oviductal function. *FSHR* expression was higher in both L‐tyrosine groups, indicating improved ovarian responsiveness to pituitary FSH, consistent with findings reported for L‐arginine (Uyanga et al. 2022) and octacosanol supplementation (Long et al. 2017). Because prolactin suppresses *FSHR* expression, the reduction in prolactin most likely served as the primary permissive signal for increased *FSHR* transcription, thereby improving follicular sensitivity to FSH and supporting hierarchy maintenance in aged hens. *LH* expression also increased in both supplemented groups, in line with Abobaker et al. (2019) and Uyanga et al. (2022). This increase may be attributed to dopamine‐mediated modulation of GnRH neurons, which enhances pituitary LH synthesis (Bana et al. 2021) and links the dopaminergic response to L‐tyrosine with the sustained laying rates observed in the present study. Although *PRL* mRNA did not decrease significantly, a tendency toward reduction was observed, which aligns with *DRD1* upregulation and previous observations with fennel seed essential oil supplementation (İpçak et al. 2025). The coordinated transcriptional profile of upregulated *DRD1*, *ESR1*, *ESR2*, *FSHR*, and *LH* with declining *PRL* is consistent with the hormonal findings and provides a molecular explanation for the improvements in laying performance and egg quality observed in this study.

Promoter CpG methylation patterns were inversely related to gene expression across all examined genes. *PRL* exhibited pronounced promoter hypermethylation in both supplemented groups, while *DRD1*, *ESR1*, *ESR2*, *FSHR*, and *LH* showed promoter hypomethylation, consistent with the corresponding transcriptional patterns. Although L‐tyrosine is not a direct methyl donor, dopamine signalling has been shown to modulate the activity of DNA methyltransferases and ten‐eleven translocation (TET) enzymes, thereby influencing promoter methylation in hormone‐responsive genes (Liu et al. 2005). The methylation changes observed here likely reflect secondary epigenetic modifications driven by L‐tyrosine–induced dopaminergic signalling rather than a direct methyl donor effect. *PRL* promoter hypermethylation, together with the observed trend toward reduced *PRL* expression, supports the hypothesis of Kumar and Biswas (1988), who linked cytosine methylation changes at the *PRL* promoter to altered transcriptional activity across physiological states. Sved et al. (1979) showed that tyrosine administration suppresses prolactin through hypothalamic dopamine synthesis, and Liu et al. (2005) demonstrated that dopamine D2 receptor activation triggers histone deacetylation at the *PRL* promoter region. Collectively, these observations suggest a mechanistic link between L‐tyrosine‐derived dopaminergic stimulation and the epigenetic silencing of *PRL* transcription. Promoter hypomethylation of *DRD1*, *ESR1*, *ESR2*, *FSHR*, and *LH* is consistent with transcriptional activation associated with promoter demethylation of estrogen receptor genes (Morshed et al. 2006; Guo et al. 2020) and steroidogenic pathway genes (Abobaker et al. 2017; Abobaker et al. 2019). Betaine supplementation has been shown to induce comparable promoter demethylation in hormone receptor genes (Omer et al. 2018; Omer et al. 2020). Zhang et al. (2023) demonstrated extensive methylome remodelling in hypothalamic‐ovarian tissues during forced moulting, further highlighting the epigenetic plasticity of the reproductive axis in laying hens. Ponsuksili et al. (2025) reported strain‐specific differences in DNA methylation of reproductive hormone pathways, indicating that epigenetic responses to nutritional interventions may vary across genetic backgrounds. Negative correlations between promoter CpG methylation and gene expression were confirmed across both supplemented groups, with hypomethylation associated with enhanced transcription and hypermethylation with transcriptional silencing. These findings suggest that L‐tyrosine operates through an integrated neuroendocrine‐epigenetic axis in aged laying hens. Enhanced dopaminergic tone may contribute to coordinated promoter methylation changes, resulting in simultaneous suppression of *PRL* and activation of key reproductive signalling genes. This integrated response aligns epigenetic, transcriptional, and hormonal profiles with sustained egg production and quality in aged laying hens. Dietary L‐tyrosine supplementation may therefore represent a targeted nutritional strategy for extending the productive phase of commercial laying flocks in the late laying phase, where age‐related dopaminergic decline constitutes a key limiting factor for sustained productivity.

## 5 Conclusions

Dietary L‐tyrosine supplementation improved laying performance, egg quality, and feed efficiency in aged laying hens, along with changes in reproductive gene expression and promoter CpG methylation. Supplementation with 1 g/kg L‐tyrosine was the most effective level and may represent a promising feed additive for commercial poultry production in late‐phase laying hens. Future research should optimise L‐tyrosine inclusion levels and evaluate responses across different laying hen strains and production systems. Further studies should also determine whether these effects persist or are heritable across production cycles.

## Ethics Statement

The authors confirm that the ethical policies of the *Journal of Animal Physiology and Animal Nutrition*, as noted on the journal's author guidelines page, have been adhered to, and that the appropriate ethical review committee approval has been received. The experimental procedures involving animals were approved by the Local Ethics Committee for Animal Experiments of Dicle University (protocol code 2022/32). The authors also confirm that all procedures complied with EU standards for the protection of animals used for scientific purposes (Directive 2010/63/EU).

## Data Availability

The data that support the findings of this study are available from the corresponding author upon reasonable request.
